# Size controllable single-crystalline Ni-rich cathodes for high-energy lithium-ion batteries

**DOI:** 10.1093/nsr/nwac226

**Published:** 2022-10-19

**Authors:** Ji-Lei Shi, Hang Sheng, Xin-Hai Meng, Xu-Dong Zhang, Dan Lei, Xiaorui Sun, Hongyi Pan, Junyang Wang, Xiqian Yu, Chunsheng Wang, Yangxing Li, Yu-Guo Guo

**Affiliations:** CAS Key Laboratory of Molecular Nanostructure and Nanotechnology, Beijing National Laboratory for Molecular Sciences (BNLMS), Institute of Chemistry, Chinese Academy of Sciences (CAS), Beijing100190, China; University of Chinese Academy of Sciences, Beijing100049, China; CAS Key Laboratory of Molecular Nanostructure and Nanotechnology, Beijing National Laboratory for Molecular Sciences (BNLMS), Institute of Chemistry, Chinese Academy of Sciences (CAS), Beijing100190, China; CAS Key Laboratory of Molecular Nanostructure and Nanotechnology, Beijing National Laboratory for Molecular Sciences (BNLMS), Institute of Chemistry, Chinese Academy of Sciences (CAS), Beijing100190, China; University of Chinese Academy of Sciences, Beijing100049, China; CAS Key Laboratory of Molecular Nanostructure and Nanotechnology, Beijing National Laboratory for Molecular Sciences (BNLMS), Institute of Chemistry, Chinese Academy of Sciences (CAS), Beijing100190, China; CAS Key Laboratory of Molecular Nanostructure and Nanotechnology, Beijing National Laboratory for Molecular Sciences (BNLMS), Institute of Chemistry, Chinese Academy of Sciences (CAS), Beijing100190, China; Beijing Advanced Innovation Center for Materials Genome Engineering, Institute of Physics, CAS, Beijing100190, China; Beijing Advanced Innovation Center for Materials Genome Engineering, Institute of Physics, CAS, Beijing100190, China; Beijing Advanced Innovation Center for Materials Genome Engineering, Institute of Physics, CAS, Beijing100190, China; Beijing Advanced Innovation Center for Materials Genome Engineering, Institute of Physics, CAS, Beijing100190, China; Department of Chemical and Biomolecular Engineering, University of Maryland, College Park, MD20742, USA; Chery New Energy Automobile Co., Ltd, Wuhu241002, China; CAS Key Laboratory of Molecular Nanostructure and Nanotechnology, Beijing National Laboratory for Molecular Sciences (BNLMS), Institute of Chemistry, Chinese Academy of Sciences (CAS), Beijing100190, China; University of Chinese Academy of Sciences, Beijing100049, China

**Keywords:** lithium-ion batteries, high energy density, Ni-rich cathodes, single-crystalline, surface energy

## Abstract

A single-crystalline Ni-rich (SCNR) cathode with a large particle size can achieve higher energy density, and is safer, than polycrystalline counterparts. However, synthesizing large SCNR cathodes (>5 μm) without compromising electrochemical performance is very challenging due to the incompatibility between Ni-rich cathodes and high temperature calcination. Herein, we introduce Vegard's Slope as a guide for rationally selecting sintering aids, and we successfully synthesize size-controlled SCNR cathodes, the largest of which can be up to 10 μm. Comprehensive theoretical calculation and experimental characterization show that sintering aids continuously migrate to the particle surface, suppress sublattice oxygen release and reduce the surface energy of the typically exposed facets, which promotes grain boundary migration and elevates calcination critical temperature. The dense SCNR cathodes, fabricated by packing of different-sized SCNR cathode particles, achieve a highest electrode press density of 3.9 g cm^−3^ and a highest volumetric energy density of 3000 Wh L^−1^. The pouch cell demonstrates a high energy density of 303 Wh kg^−1^, 730 Wh L^−1^ and 76% capacity retention after 1200 cycles. SCNR cathodes with an optimized particle size distribution can meet the requirements for both electric vehicles and portable devices. Furthermore, the principle for controlling the growth of SCNR particles can be widely applied when synthesizing other materials for Li-ion, Na-ion and K-ion batteries.

## Teaser

Controllable grain sizes in a wide range enable the single-crystalline Ni-rich cathode to break through the bottleneck of volumetric energy density, which makes it qualified to replace LiCoO_2_ and alleviate the cobalt crisis.

## INTRODUCTION

Since the commercialization of lithium-ion batteries (LIBs) in the 1990s, LiCoO_2_ has been considered the first choice in cathode materials, especially in ‘3C’ products (computers, communications and consumer electronics), due to the fact that it has the highest volumetric energy density among commercially available cathode materials (Fig. S1a and b) [1–4]. However, due to scarce resources, high cost and the severe environmental pollution caused by cobalt, Ni-rich cathodes have attracted a lot of attention as a more sustainable choice [5–8]. Ni-rich cathodes have a high specific energy and are cost-effective, making them suitable for electric vehicle application [9–11]. However, they fail to be applied to 3C products due to their lower volumetric energy density from a lower packing density compared to LiCoO_2_ (Fig. S1c and d) [12–14].

Single crystallization is a smart strategy that can improve not only safety by alleviating interface degradation but also energy density via increasing electrode press density [15–18]. However, the currently available single-crystalline Ni-rich (SCNR) cathodes have a typical particle size of <4 μm, which means the press density is unsatisfactory. To further improve the press density of the Ni-rich cathodes benchmarking LiCoO_2_, a large single crystal is necessary to fully reduce voids. Generally, increasing calcination temperature is the most common method to promote crystal growth, but it may incur oxygen escape from the host structure, the reduction of partial Ni^3+^ and consequent formation of the non-stoichiometric phase, which will ultimately deteriorate the electrochemical performance [19–22]. Therefore, a relatively low calcination temperature (<800°C) is generally preferred when synthesizing Ni-rich cathodes so that super-large SCNR cathodes are unavailable to guarantee structure integrity and electrochemical performance [20,23]. Although the molten-salt-assisted method can be used to synthesize SCNR cathodes, it tends to induce impurity phases and needs an additional water washing process to remove the residual salt, which severely reduces the cycle stability of the materials [17,24,25]. Furthermore, the large SCNR cathodes have a long-range ordered crystalline structure, which is likely to cause lattice collapse after a large amount of Li^+^ is extracted, consequently reducing the material's rate capability and cycle stability [26]. Thus, synthesis of large SCNR cathodes (>5 μm) is very challenging [27].

Here, we have successfully prepared a series of SCNR cathode materials with controllable particle sizes in a range of 0.5–10 μm by using trace amounts of sintering aids Al_2_O_3_ and CeO_2_. The introduction of CeO_2_ dramatically promotes crystal growth by adjusting ion mobility and surface energy, which allows a larger particle size. Meanwhile, spectroscopic and structural characterizations reveal that Ce^4+^ is enriched on the surface and Al^3+^ distributes homogeneously in the bulk, enhancing the lattice and interface stability synergistically. The as-prepared SCNR cathode exhibits excellent electrochemical performance, and its available high press density enables a 4 Ah pouch cell delivering impressive energy densities of 303 Wh kg^−1^ and 730 Wh L^−1^, at a level that competes with state-of-the-art LIBs using LiCoO_2_ cathodes. Thus, the SCNR cathode qualifies as an alternative to LiCoO_2_, and is bound to alleviate cobalt reliance, contributing to the development of sustainable LIBs.

## RESULTS

### Optimization of sintering aids to promote crystal growth

**Figure 1.**
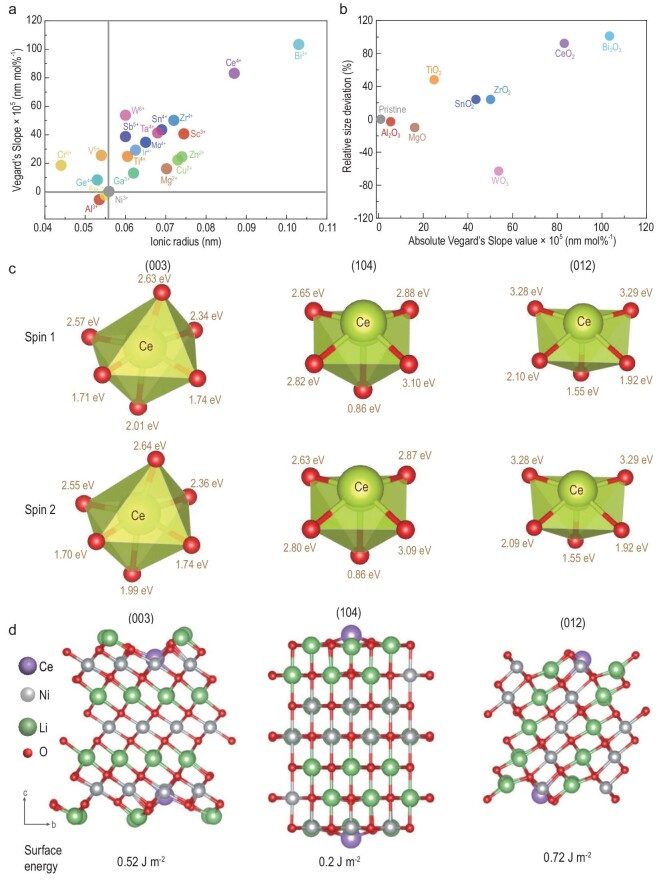
Sintering aids effect on crystal growth. (a) Vegard's Slope vs. ionic radius for possible sintering aids. (b) The relative particle size deviation of SCNR materials synthesized with different sintering aids compared with pristine NCM materials. The size deviation is the size difference between the cathodes calcined with varied sintering aids and the bare cathode. (c) The bond strength between surface transition metal Ce and oxygen atoms, obtained by crystal orbital Hamilton population (COHP) analysis. (d) The surface energy of typical exposed facets in Ni-rich cathode materials (003), (104) and (012) after the introduction of Ce^4+^.

Crystal growth at relatively low calcination temperature can be regulated using sintering aids, which can be rationalized via Vegard's Slope [28]. The sintering aids that do not match the host will have a large absolute Vegard's Slope. They are difficult to incorporate into the host crystal lattice, leading to enrichment on the particle surface instead [29]. Some concentrated dopants on the surface of the particles may reach a specific ratio and form a eutectic film with a melting point below that of the two pure substances. This thin film promotes interface atomic diffusion even below the melting point [30]. In contrast, the ions that match with the host with a small absolute Vegard's Slope can be incorporated into the crystal lattice, which has less impact on the growth of SCNR cathodes. The cations with different Vegard's Slopes were selected as sintering aids to synthesize the SCNR particles (Fig. 1a). The size deviation of the SCNR particles correlates well with Vegard's Slope difference, and CeO_2_ and Bi_2_O_3_ indeed promote crystal growth of SCNR cathodes (Fig. 1b and Fig. S2). Compared with Ce^4+^, the low valence of Bi^3+^ cannot ameliorate the kinetic hindrance in the large SCNR cathode, leading to an inferior performance (Fig. S3). Finally, we chose CeO_2_ to obtain SCNR cathodes, simultaneously adding Al_2_O_3_ for its well-known function of stabilizing cathode structure.

### Crystal growth mechanism

To understand the critical role of sintering aids in promoting crystal growth, the TM-O bond energy and surface energy were calculated based on the typically exposed facets (003), (104) and (012) of Ni-rich cathodes [31]. In solid-state synthesis, the mass transfer for crystal growth is dominated by grain boundary migration and the rate ${V}_B$ can be demonstrated via the following equation, which is the product of mobility ${M}_B$ and driving force $\Delta p$ [20]:

$$\begin{eqnarray*}
{V}_B &=& - {M}_B\Delta P,\\
{M}_B &=& \frac{{{f}_a{a}^4}}{{{k}_BT}}{\rm{exp}}\left[ {\frac{{ - {E}_A}}{{{R}_gT}}} \right],\\
\Delta P &=& g\Delta \gamma \left( {\frac{1}{{{G}_1}} - \frac{1}{{{G}_2}}} \right),
\end{eqnarray*}$$

where ${k}_B$ and ${R}_g$ are the Boltzmann constant and gas constant, respectively. Besides the intrinsic atomic size *a* and vibration frequency ${f}_a$, temperature *T* and activation energy ${E}_A$ account for a prominent influence on ${M}_B$ with an Arrhenius relation, which means raising the temperature can accelerate grain growth dramatically. Ce-O bonds have higher bond energy than Ni-O bonds in the above three facets (Fig. 1c and Fig. S4a), which can stabilize lattice oxygen atoms and suppress the release of oxygen from the sublattice at a high temperature. Thus, the enhanced Ce-O bonds will allow a higher calcination temperature *T* for the cathodes with Ce^4+^ doping. On the other hand, the driving force $\Delta P$ lies in surface energy difference $\Delta \gamma $ and the discrepancy of sizes ${G}_1$, ${G}_2$ between adjacent grains. *g* is a geometric factor dependent on grain shape. Specifically, small grains are prone to coalesce into large grains so that surface energy can be reduced due to a lower specific surface area, similar to Ostwald ripening. Ce^4+^-doped SCNR cathodes show lower surface energy than those without Ce^4+^ doping in all three surfaces (Fig. 1d and Fig. S4b), so CeO_2_ is thermodynamically favored on the surface of SCNR particles. The segregation of Ce^4+^ on the particle surface will further reduce the surface energy (namely increase $\Delta \gamma $), so Ce^4+^ will promote crystal growth to reach a thermodynamically stable state.

### Structure of SCNR cathodes

**Figure 2.**
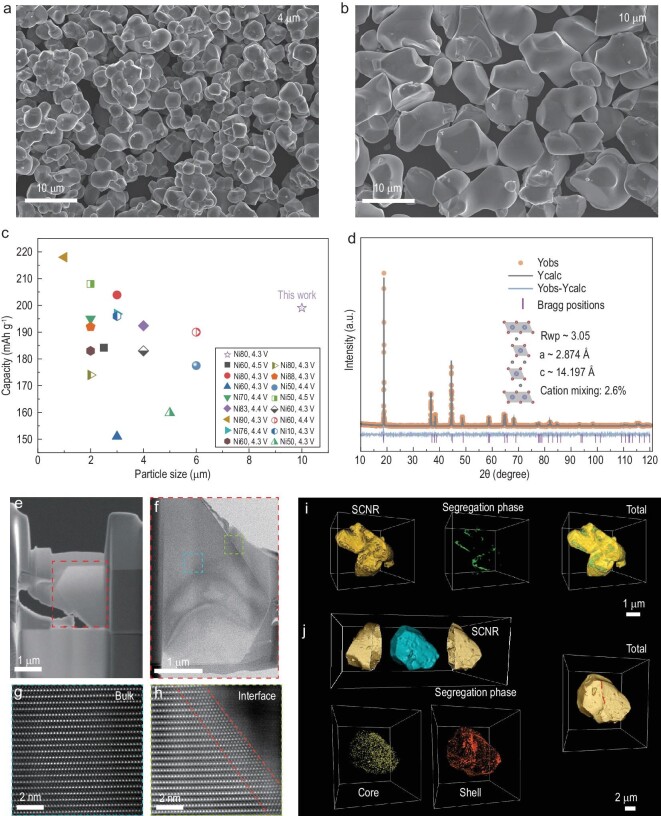
The morphology, structure and phase distribution of SCNR cathodes. SEM images of SCNR materials with a particle size of (a) 4 μm and (b) 10 μm. The scale bars are 10 μm. (c) Particle size and capacity comparison of the SCNR cathode from this work and high capacity single-crystalline NCM cathodes from the literature. (d) Rietveld refinement of an SCNR cathode with a particle size of 10 μm. (e and f) SEM image and low-magnified STEM image of an SCNR cathode sample prepared by FIB. (g and h) High-resolution STEM images of an SCNR cathode. (i and j) 3D spatial distribution of different phases in SCNR particles, probed by nano-CT.

By employing CeO_2_ and Al_2_O_3_ as sintering aids, we synthesized SCNR cathode particles with sizes ranging from 500 nm to 10 μm by adjusting calcination temperature, and the crystal grains are smooth without visible residual alkali salts (Fig. 2a and b, and Fig. S5a–f). As shown in Fig. 2c and Table S1, we synthesized 10 μm SCNR cathode particles, the largest Ni-rich single-crystalline cathode reported to date. In addition, all SCNR cathode particles were prepared at the pilot scale (Fig. S5g and h). According to powder X-ray diffraction (XRD), SCNR cathode materials conform to the typical *R*-3*m* layered structure without any impurity phases. More specifically, the Rietveld refinement analysis for the XRD pattern of the large SCNR sample (10 μm) synthesized at 950^o^C yields lattice parameters of *a* = *b* = 2.874 Å, *c* = 14.197 Å with a Li/Ni cation mixing of 2.6% (Fig. 2d). Such a low level of cation mixing indicates a well-preserved layered structure at a calcination temperature of 950^o^C due to the adding of sintering aids CeO_2_ and Al_2_O_3_.

The crystal structure of a 10 μm SCNR cathode at atomic scale was characterized using spherical aberration-corrected scanning transmission electron microscope (STEM) imaging with focused ion beam (FIB) preparation (Fig. 2e). The cross-section image demonstrates that the bulk of the particle is dense without any void or crack (Fig. 2f). Atomic-resolution STEM results further verify that the bulk layered structure does not have obvious cation-mixing (Fig. 2g), while a thin layer of rock-salt phase can be found on the particle surface (Fig. 2h), which may be attributed to the surface enrichment of Ce^4+^.

To verify the promoting mechanism of crystal growth, nanoscale X-ray computed tomography (nano-CT) was utilized to resolve the spatial distribution of the trace Ce^4+^ in two SCNR particles with sizes 3 μm and 10 μm. The process is shown in detail in Fig. S6. Both SCNR cathode materials are single-crystalline particles with a small amount of segregation phase shell on the particle surface. The segregation phase for the 10 μm particle is about twice the thickness of that for the 3 μm one (Fig. S7a). On the 3 μm particle, the surface segregation phase is sparsely distributed and locally enriched (Fig. 2i). However, a dense and well-connected surface segregation phase covers the 10 μm particle (Fig. 2j). Statistically, the segregation phase on the 10 μm particle shell has a larger average volume and a wider volume distribution than that on the 3 μm particle, demonstrating its densely covered morphology (Fig. S7b and c). The different Ce^4+^ distribution in the SCNR particles of two sizes indicates an unambiguous atomic diffusion tendency in the sintering process. As the grain grows, Ce^4+^ ions continuously migrate to the particle surface. Meanwhile, the gradually intensified surface Ce^4+^ enrichment further drives mass transfer for crystal growth under thermodynamic momentum, which validates the potential mechanism of the particle size growth.

### Performance of SCNR cathodes

The electrochemical performance of SCNR cathode materials with varying grain sizes is evaluated using half-cells (Fig. S8a–e). While the initial charge capacity is almost the same (Fig. S8f), the specific discharge capacity decreases from 210 to 191 mAh g^−1^ as the particle size increases from 500 nm to 10 μm. This phenomenon indicates the poorer lithium diffusion kinetics of the larger SCNR particles, which is in agreement with Fick's first law of diffusion (*τ_eq_ ∼ L*^2^/2*D*, where *τ_eq_* is diffusion time, *D* is the diffusion coefficient and *L* is diffusion length) [32]. When the charge-discharge rate is reduced to 0.01 C, the specific discharge capacity of the 10 μm SCNR cathode is restored to 205 mAh g^−1^, which is close to that of the 500 nm cathode, suggesting that the capacity loss resulting from the sluggish reinsertion of Li^+^ is reversible (Fig. S8g and h). We also compare the first charge-discharge curves of pristine single crystals, polycrystals, single crystals synthesized with different sintering aids (Fig. S9a–i). It can be seen that most modified single-crystal samples exhibit similar capacity to the pristine ones.

**Figure 3.**
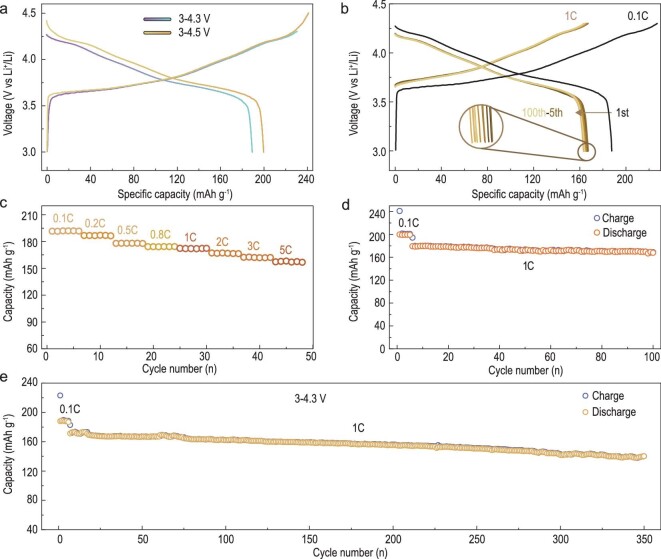
Electrochemical performance of SCNR cathodes with a particle size of 10 μm in coin-cells. (a) The first charge-discharge curves of the SCNR half-cell at charge cut-off voltages of 4.3 and 4.5 V. (b) The charge-discharge curves of the SCNR half-cell for the first 100 cycles. For the first five cycles, the cell was tested at 0.1C, and then at 1C for subsequent cycles. (c) Rate performance of the SCNR half-cell. The charge and discharge rate before 1C is the same, and the charge rate is 1C with discharge rate varying from 1C to 5C. (d and e) Cycle performance of the SCNR cells at charge cut-off voltages of 4.5 and 4.3 V.

Given that a large SCNR cathode is essential for increasing volumetric energy density, we then focused on testing a 10 μm SCNR cathode in half-cells to evaluate its application potential for LIBs. The cathode delivers a high specific discharge capacity of 191 and 200 mAh g^−1^ at 0.1C with a charging cut-off voltage of 4.3 and 4.5 V respectively (Fig. 3a). Meanwhile, there is negligible voltage polarization of <0.01 V observed in the charge-discharge curves after 100 cycles at 1C, which can be attributed to the stable interface during repeated cycling (Fig. 3b). The SCNR cathode also demonstrates a good rate capability, preserving a discharge capacity of around 150 mAh g^−1^ even under a high rate of 5C (Fig. 3c). The exceptional cycling stability in the voltage range of 3–4.3 V with a capacity retention of 80.4% after 350 cycles is also demonstrated (Fig. 3e). When the charging cut-off voltage is increased to 4.5 V, the capacity still maintains 94.4% after 100 cycles (Fig. 3d).

The electrochemical performance of LIBs is closely related to the evolution of the cathode-electrolyte interphase (CEI) during cycling. *Ex-situ* X-ray photoelectron spectroscopy (XPS) and electrochemical impedance spectroscopy (EIS) are performed to reveal the CEI evolution process of SCNR cathodes. The evolution of F 1*s*, C 1*s* and O 1*s* spectra for different cycles (Fig. S10) suggests that the CEI relevant components of LiF, C=O, C−O and C−H, increase distinctly before 20 cycles. Then there is only a slight increase of these CEI species in the subsequent cycles, confirming the stability of the CEI. In terms of the electrode kinetics as shown in EIS (Fig. S11a), there is little change in charge-transfer resistance for the first 100 cycles, which also indicates that a stable CEI was formed on the cathode during cycling. This observation can be likely attributed to the beneficial Ce^4+^-enriched nanoscale rock-salt layer on the surface of the SCNR sample, which acts as a surface protective layer for the cathode and stabilizes the CEI.

The thermal and structural stability of cathode materials is a critical factor for battery safety, and important for practical application. The 3 μm SCNR cathode sample is much more thermally stable compared to the reported agglomerated polycrystalline NCM811 with spherical particles. More importantly, when the particle size of an SCNR cathode increases from 3 to 10 μm, the thermal decomposition temperature further increases from 280.8°C to 286.8°C, whilst the exothermic heat is reduced from 1463 J g^−1^ to 982.9 J g^−1^, demonstrating the great benefit of designing large SCNR cathodes for the safety enhancement of LIBs (Fig. S11b). The *in-situ* XRD results in Fig. S11c show excellent structural stability. The (003) peak gradually shifts to a lower angle with the increase in operating voltage, but it moves reversely to the higher angle side when the charging voltage is higher than 4.1 V, suggesting that the lattice parameter *c* expands first but then drops during the Li^+^ extraction process.

**Figure 4.**
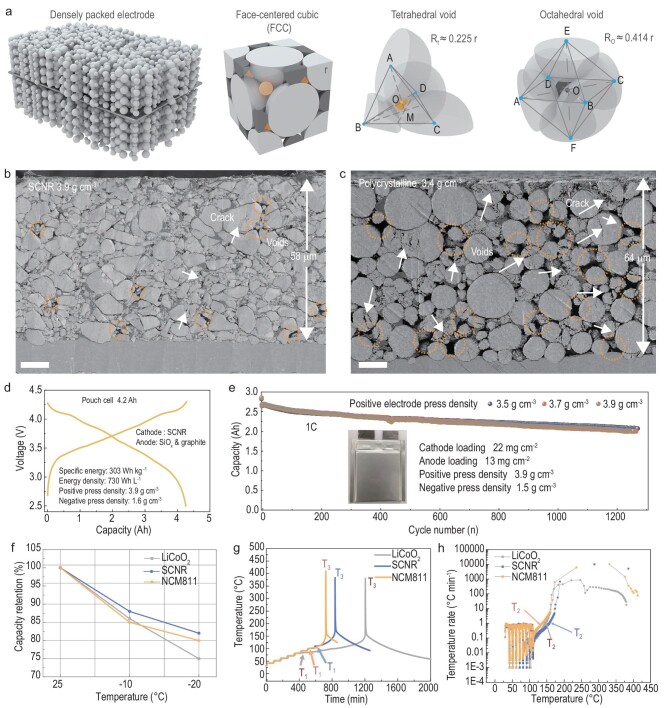
Characterizations of the SCNR material under practical application conditions. (a) Schematic illustration of the dense electrode, four octahedral voids and eight tetrahedral voids in each unit cell. (b and c) Cross-section SEM images of SCNR and polycrystalline spherical NCM811 electrodes with the same capacity of 4.5 mAh cm^−2^. The scale bars are 10 μm. (d) Charge and discharge curves of a SCNR|SiO_x_&graphite pouch cell. (e) Cycle performance of SCNR|graphite pouch cells. (f) A comparison of LiCoO_2_, SCNR and polycrystalline spherical NCM811 cathodes in pouch cells under low temperature. (g and h) A comparison of T-t curve and temperature rates of the LiCoO_2_, SCNR and polycrystalline spherical NCM811 cathodes in pouch cells.

The SCNR cathode was also evaluated in commercial pouch cells using commercial graphite or SiO_x_/graphite anodes. To further improve electrode tapping density, we filled packing voids among large particles with smaller ones. For simplicity, we take face-centered cubic (FCC) stacking of spherical particles as an example here. If the octahedral and tetrahedral voids in the model are filled with spherical particles of appropriate sizes, the space utilization can further increase from 74% to 81% (Fig. 4a). In practice, a high loading of active materials up to ∼22 mg cm^−2^ (single-side ∼4.5 mAh cm^−2^) is achieved, and the electrodes can reach slightly variable press densities by adjusting roll design (Fig. S11d–g). A press density of 3.9 g cm^−3^ is achieved, which is much higher than that of the electrode made by the commercial NCM cathode (3.4 g cm^−3^) and is also close to that of the LiCoO_2_ cathode (4.1 g cm^−3^). To the best of our knowledge, the press density 3.9 g cm^−3^ is the highest reported for NCM cathode materials. The cross-section images clearly demonstrate the difference in void and crack distribution in the two kinds of densely packed electrodes (Fig. 4b and c).

Two types of pouch cells are assembled to evaluate the battery performance. The designed capacity of a single pouch cell is 2.9 Ah (vs. graphite, press density 1.5 g cm^−3^) and 4 Ah (vs. SiO_x_ and graphite, capacity 500 mAh g^−1^, press density 1.6 g cm^−3^). The 4 Ah pouch cell exhibits both a high specific gravimetric energy density of 303 Wh kg^−1^ and a high volumetric energy density of 730 Wh L^−1^ (based on all components of the pouch cell) (Fig. 4d and Table S2). The 2.9 Ah pouch cell exhibits remarkable cycle stability with a capacity retention of 76% after 1200 cycles (Fig. 4e), which is comparable with LiCoO_2_-based commercialized Li-ion batteries. Furthermore, the SCNR pouch cell possesses impressive low-temperature performance. Taking the capacity at 1C with a cut-off voltage of 4.25 V in room temperature as a benchmark, the SCNR cell exhibits 88% and 83% capacity retention in −10°C and −20°C respectively, which are significantly higher than NCM811 and LiCoO_2_ (Fig. 4f). The thermal runaway behavior of SCNR pouch cells is also investigated. Taking NCM811 and LiCoO_2_ cathodes for comparison, we employ accelerating rate calorimetry (ARC) with a standard process of heat-wait-seek to acquire the accurate thermal runaway behavior in pouch cells (Fig. 4g and h, and Table S3). The onset temperature (T_1_, abnormal heat generation) and triggering temperature (T_2_, tipping point of sharp temperature increase) of the SCNR pouch cell is 92°C and 157°C respectively, which are higher than those of LiCoO_2_ (72°C and 146°C) and NCM811 (83°C and 136°C). The results suggest that the thermal stability of large SCNR cathodes is superior to both polycrystalline NCM and single-crystalline LiCoO_2_.

## DISCUSSION

**Figure 5.**
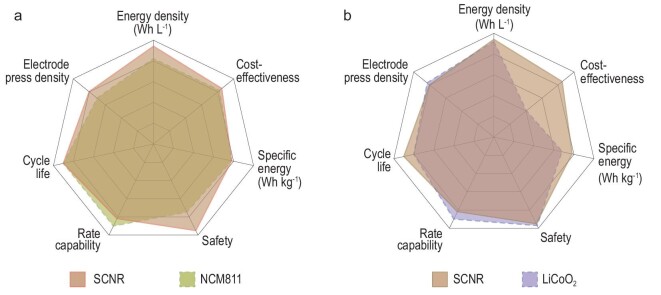
Benchmarking SCNR cathodes against (a) polycrystalline spherical NCM811 and (b) LiCoO_2_. The Radar maps compare the SCNR cathode with polycrystalline NCM811 and LiCoO_2_ in terms of several key parameters that are critical for practical application.

The comprehensive battery performance of the SCNR cathode is compared to commercial polycrystalline NCM811 and LiCoO_2_ in the form of a Radar map (Fig. 5 and Tables S4–S6). Compared to NCM811, an SCNR cathode exhibits particularly outstanding safety and energy density besides other key parameters (Fig. 5a). The SCNR cathode has a similar particle size to typical commercial polycrystalline NCM811, but the grain boundaries are eliminated. Therefore, the detrimental side reactions and gaps can be reduced due to the lower specific surface area. In contrast with LiCoO_2_, both cathodes possess the dense characteristic of a single crystal, so the SCNR cathode is able to have nearly the same energy density and level of safety as LiCoO_2_. The most prominent advantage is the cost competitiveness of the SCNR cathode (Fig. 5b), which makes it very attractive as a substitute for LiCoO_2_ in 3C applications; this may relieve the strain on cobalt resources in the next century [6,33–35]. It is important to note that the intrinsic drawbacks of Ni-rich cathodes, like charge heterogeneity and cracking, can still exist, and will be prominent especially for their operation in high temperatures. When temperature rises, the ions can overcome the reaction barrier more easily, causing much more severe internal stress evolution, and the reaction rate between Ni^4+^ and electrolyte can be increased dramatically. The interplay among lattice and surface defects, charge heterogeneity, and high temperature cycle stability is a frontier research topic that is worth systematic follow-up efforts.

In conclusion, it is not hard to acquire large-size SCNR cathodes; this can be achieved just by elevating calcination temperature. However, the electrochemical performance of SCNR cathodes obtained in that way are not competitive with their polycrystalline counterparts and LiCoO_2_. A high temperature can endow ions with enough mobility to overcome barriers between grain boundaries, but it comes at a cost: the destruction of the layered structure and severe cation disordering. The adding of CeO_2_ in this work accelerates the mass transfer along grain boundaries via a micro-liquid environment around grains, and enhances surface energy drive, promoting crystal growth both kinetically and thermodynamically. Also, Ce^4+^ ions segregate on the particle surface to construct a stubborn rock-salt layer, and Al^3+^ is simultaneously incorporated into the bulk lattice, enhancing the layered structure. Benefiting from the synergistic effect, the SCNR cathode is able to successfully grow without disturbing the electrochemical performance too much. It is noteworthy that the selection of sintering aids is not unique; this can be further explored by referring to Vegard's Slope. Our findings provide the methodology with which to synthesize a large SCNR NCM cathode with superior battery performance. This is very promising with regard to alleviating the sustainability issue surrounding cobalt resources.

## Supplementary Material

nwac226_Supplemental_File
