# Supplementary material for: Size controllable single-crystalline Ni-rich cathodes for high-energy lithium-ion batteries
Source: Natl Sci Rev. 2022 Oct 19;10(2):nwac226. doi: 10.1093/nsr/nwac226 (PMC9935991; doi:10.1093/nsr/nwac226)
Supplement: nwac226_Supplemental_File [file nwac226_supplemental_file.docx]

**Supplementary data**

**Size controllable single-crystalline Ni-rich cathode for high-energy lithium-ion batteries**

Ji-Lei Shi^1,5,†^, Hang Sheng^1,†^, Xin-Hai Meng^1,5,†^, Xu-Dong Zhang^1^, Dan Lei^1^, Xiaorui Sun^2^, Hongyi Pan^2^, Junyang Wang^2^, Xiqian Yu^2,^*, Chunsheng Wang^4,^*, Yangxing Li^3,^* and Yu-Guo Guo^1,5,^*

^1^CAS Key Laboratory of Molecular Nanostructure and Nanotechnology, Beijing National Laboratory for Molecular Sciences (BNLMs), Institute of Chemistry, Chinese Academy of Sciences (CAS), Beijing 100190, China;

^2^Beijing Advanced Innovation Center for Materials Genome Engineering, Institute of Physics, CAS, Beijing 100190, China;

^3^Chery New Energy Automobile Co., Ltd, Wuhu 241002, China;

^4^Department of Chemical and Biomolecular Engineering, University of Maryland, College Park, MD 20742, USA;

^5^University of Chinese Academy of Sciences, Beijing 100049, China

^†^Equally contributed to this work.

***Corresponding authors.** E-mails: ygguo@iccas.ac.cn; xyu@iphy.ac.cn; cswang@umd.edu; liyangxing@mychery.com

**Methods**

**Synthesis of SCNR cathode materials.** The co-precipitation method was used to synthesize the SCNR precursor Ni_0.8_Co_0.1_Mn_0.1_(OH)_2_, and the particle size of the precursor was controlled to be less than 4 μm. The Ni_0.8_Co_0.1_Mn_0.1_(OH)_2_ precursor and LiOH·H_2_O were uniformly mixed with nano-Al_2_O_3_ and nano-CeO_2_. The molar ratio of LiOH:TM (transition metal) is 1.05 and the amounts of both Al_2_O_3_ and CeO_2_ are 0.4 mol%. The mixture was first calcined at 500 ℃ for 5 hours, and mixed again after naturally cooling down to the room temperature. After that, the mixture was calcined again for 5 hours and the temperature varies for different targeted particle size (10 μm: 950 ℃; 8 μm: 930 ℃; 6 μm: 900 ℃; 4 μm: 870 ℃; 2 μm: 850 ℃), and then the mixture was annealed at 780 ℃ for 10 hours.

**Coin cell tests**: The positive electrodes were prepared by mixing active materials with carbon black and poly-vinylidene fluoride (PVDF) in *N*-methylpyrrolidone (NMP) as the solvent in a weight ratio of 90:5:5 for coin cells and then coating the slurry onto Al foil followed by vacuum drying at 80 °C for 12 h. The electrode discs were punched from the foil and weighed. The range of active material loading was ~10 mg cm^-2^. Type 2032 coin cells were assembled by using Li metal as the negative electrode, Celgard PP/PE/PP film as separator and 1 M LiPF_6_ dissolved in EC/DEC/DMC (1:1:1) carbonate as electrolyte in an argon-filled glove box. Galvanostatic discharge/charge cycles and rate tests were performed on a LAND system for coin cells. Electrochemical impedance spectroscopy (EIS) with frequency varying from 100 kHz to 0.1 Hz was obtained by using a PARSTAT (VMC).

**Pouch cell tests**: The positive electrode was made of 97.5% active materials, 0.75% conductive Super P, 0.75% multi-walled carbon nanotube and 1% PVDF binder, while the anode electrode was made of 94% graphite, 2% conductive carbon and 4% carboxymethyl cellulose/polymerized styrene-butadiene rubber (CMC-SBR) as a binder. The single-sided mass loading of SCNR was ~22 mg cm^-2^ and that of graphite anode was ~14 mg cm^-2^, corresponding to an N/P ratio of 1.1. The composite electrode was dried in a vacuum for 48 h and then rolled to a press density of 3.9 g cm^-3^ for cathode, 1.5 g cm^-3^ and 1.6 g cm^-3^ for the graphite and SiO_x_ & graphite anode, respectively. Two pouch cells of 2.9 Ah and 4 Ah were assembled with the mold-cut electrodes and an electrolyte to capacity ratio of 2.5 g Ah^-1^. The charge/discharge tests of pouch cells were performed on a NEWARE CT-4008 system at 25 ^o^C. After formation cycles at a low rate, subsequent cycles were carried out in the procedure that the cells were charged to 4.25 V at 1C and held at 4.25 V until the current dropped below 0.1C. Then they were discharged to 2.8 V at 1C. Comparison sample polycrystalline NCM811 (3-4.25V, specific capacity~201 mAh g^-1^, electrode press density 3.4 g cm^-3^) and LiCoO_2_ (3-4.4V, specific capacity~180 mAh g^-1^, electrode press density 4.15g cm^-3^) are purchased from MGL New Materials Co., Ltd.

**First Principles Calculations：**Calculations based on the density functional theory (DFT) in this work were implemented using the Vienna Ab initio Simulation Package (VASP). The generalized gradient approximation (GGA) method developed by Perdew, Burke, and Ernzerhof (PBE) was adopted to describe the exchange correlation function. The DFT+U method was used to the correct the localized effect of d and f electrons. The U_eff_ (=U-J) values for Ni 3d electrons and Ce 4f electrons were set to 6.2 and 5.3 eV, respectively. The cut-off energy was set to 520 eV with a k-point spacing less than 0.3 Å^-1^. All structures were relaxed thoroughly with the energy convergence criterion of 10^−5^ eV/atom and force convergence criterion of 0.01 eV/Å. The tetrahedron method with Blochl corrections was used to accurately calculate the ground state energy.

**Surface energy:** Three low-index facets were selected in this work, namely (104), (003) and (012). For (104) facet, it is a nonpolar surface. There is only one kind of atomic arrangement for the outmost atoms. For (003) and (012) facets, they are polar surfaces. There are different terminations. To reduce the cost of computation, 50% Li-covered termination for (003) facet and 50% O-covered termination for (012) facet were construct referring to previous work. Besides, the symmetric slabs were adopted to avoid the influence of dipole moments. Based on the above criteria, all cells for surface calculations consisted of a multiple-layers slab and a 15 Å vacuum layer. The slabs contain 168, 120 and 144 atoms for (104), (003) and (012) surface models, respectively. All of them satisfy the stoichiometric ratio. Two Ni atoms located at equivalent positions in the upper and lower slabs were replaced with Ce atoms to construct the Ce-doped systems and the chemical formula can be written uniformly as LiNi_1-x_Ce_x_O_2_ (x=0.0476, 0.0556 and 0.0667 for (104), (003) and (012) surface, respectively). The surface energy can be represented as:

$$\boldsymbol{\gamma=}\frac{\boldsymbol{E}_{\boldsymbol{surf}}\boldsymbol{-}\boldsymbol{E}_{\boldsymbol{bulk}}}{\boldsymbol{2}\boldsymbol{A}}$$

where $\boldsymbol{E}_{\boldsymbol{surf}}$ is the total energy of the surface model, $\boldsymbol{E}_{\boldsymbol{bulk}}$ is the total energy of the bulk structure, and A is the surface area. For Ce-doped system, it is difficult to construct the bulk structures with the same doping ratio as the three slabs directly, we constructed bulk phase structures with different doping ratios and calculated their total energy. $\boldsymbol{E}_{\boldsymbol{bulk}}$ varies linearly with x, so we can obtain the $\boldsymbol{E}_{\boldsymbol{bulk}}$ corresponding to each Ce-doped surface model by using linear interpolation.

**Bond strength Analysis:** To investigate the effect of Ce^4+^ doping on surface oxygen atoms, it was necessary to analyze the bond strength between surface transition metal (Ni or Ce) and oxygen atoms, which could be obtained by crystal orbital Hamilton population (COHP) analysis. After completing the DFT calculations, the ICOHP (integration of COHP up to fermi energy) calculations could be succeeded using LOBSTER software.

**SEM:** The surface morphology of the materials was observed by scanning electron microscopy (SEM, JEOL 6701).

**XRD and *in-situ* XRD:** The X-ray diffraction (XRD) measurements were conducted using a Bruker (D8 ADVANCE) diffractometer with Cu Kα radiation (λ = 1.5405 Å); the scan range for powder is 10-120°(*2θ*) and for *in-situ* measurements is 10-80°(*2θ*). A specially designed Swagelok cell was used for the *in-situ* XRD experiments and X-ray-transparent ultra-thin aluminum (12 μm) was used as a test window. The *in-situ* XRD patterns were collected every 30 min with the cell operating at a current of 0.1C (1C= 200 mA g^-1^) The Rietveld refinement method was conducted using TOPAS program.

**Nano-CT:** Nanoscale X-ray computed tomography (Nano-CT) was employed using ZEISS Xradia 800 Ultra with 8 keV Cu Kα X-ray source. SCNR cathode particles with average particle sizes of 3 μm and 10 μm were glued onto the top of pin holders. In order to enhance reconstruction quality, all samples were first placed inside the operation chamber for 12 h before projection acquisition to equilibrate the temperature variation, which might cause sample motion during detection. Tomography was scanned at the high-resolution (HRES) mode with a 10 μm Ni filter to decrease background noises. The voxel size was 32 nm, actual spatial resolution was about 90 nm, and the corresponding field of view was 15×15 μm^2^. For tomographic measurements here, 901 projections were collected over a 180° rotation (0.2°/scan) with a 2 by 2 binning and an exposure time of 60 s (3 μm) and 70 s (10 μm), respectively. Tomography was reconstructed using XMR econstructor (Carl Zeiss Inc.). 3D volume rendering, image segmentation and statistical analysis were performed by Dragonfly Pro (Object Research Systems Inc.).

**STEM:** The high-resolution spherical aberration scanning transmission electron microscope (STEM) samples were prepared by using a focused-ion beam microscope (Helios NanoLab 600i). STEM images were obtained in a scanning transmission electron microscope (JEOL, Tokyo, Japan) with a double-hexapole Cs correctors (CEOS GmbH, Heidelberg, Germany) at operating voltage 200 kV and the spatial resolution of the microscope was defined by the probe forming an objective lens to be better than 80 picometres.

**XPS:** X-ray photoelectron spectroscopy (XPS) measurements were performed through Kratos Axis Supra instrument with a monochromated Al Kα source (1486.6 eV) operating at 300 W under base pressure of 8×10^-10^ Torr. An aperture of 110 μm and pass energy of 40 eV were used to acquire narrow spectra of the C 1*s*, O 1*s*, F 1*s*, and Ni 2*p*. All data were collected using charge compensation with a low-energy electron gun in order to achieve homogenous surface potential. XPS data were analysed using the ESCApe XPS software and linear background correction model. The hydrocarbon C 1*s* line at 284.8 eV was used for all spectra referencing. Parallel imaging across a 200 × 200 μm field of view was performed using the imaging XPS mode with a pass energy of 160 eV.

**DSC:** The cells were charged to 4.3 V (vs Li^+^/Li) and disassembled in an Ar-filled box for the differential scanning calorimetry (DSC) tests. The measurements were carried out in a differential scanning calorimeter (NETZSCH) by using a stainless-steel sealed pan with 15-20 mg samples (including solids and electrolyte) and the scanning rate was 10 °C min^-1^.

**ARC:** The ARC tests were performed in an Extended Volume ARC (EV-ARC, Thermal Hazard Technologies) by a heat-wait-seek (HWS) mode, the cells (charged to 100% SOC) would be heated by 10 ℃ followed by 1 hour wait, the process would be repeated until T1, the temperature when self-heating rate ≥ 0.02 ℃ min^-1^.


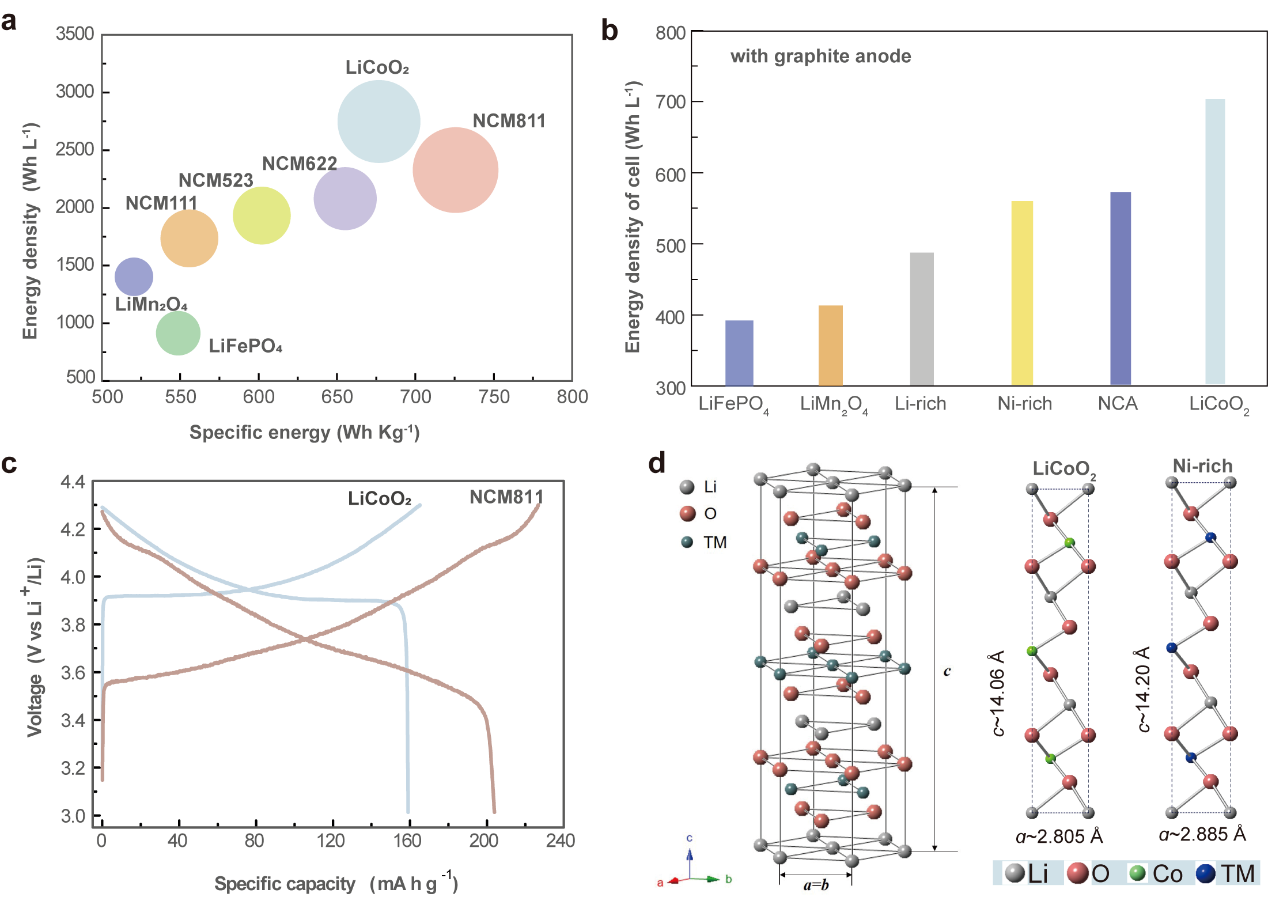


**Figure S1.** Energy density comparison of the commercialized cathode materials. **a,b,** Energy density of commercialized cathode materials and corresponding lithium-ion batteries**. c,** Charge and discharge curves of commercialized LiCoO_2_ and Ni-rich NCM811. **d,** Comparison of the cell parameters of LiCoO_2_ and Ni-rich cathode materials. The volume energy density of electrode materials (*E_v_*) depends on the specific capacity (*C*), average output voltage (*V*) and press density (*ρ*), which can be expressed as *Ev*=*C*×*V*×*ρ*. The available capacity of LiCoO_2_ within the stable electrochemical window of carbonate electrolytes (usually 4.3 V *vs* Li^+^/Li) is merely limited at ~160 mA h g^–1^, while that of Ni-rich NCM811 material is ~200 mAh g^–1^. The average voltage of LiCoO_2_ is 0.1 V higher than that of Ni-rich NCM811 (LiCoO_2_ is 3.9 V and NCM811 is 3.8 V vs Li^+^/Li). The lattice volume of LiCoO_2_ is smaller than that of Ni-rich NCM, which results in the higher crystal density of LiCoO_2_ than that of Ni-rich NCM.


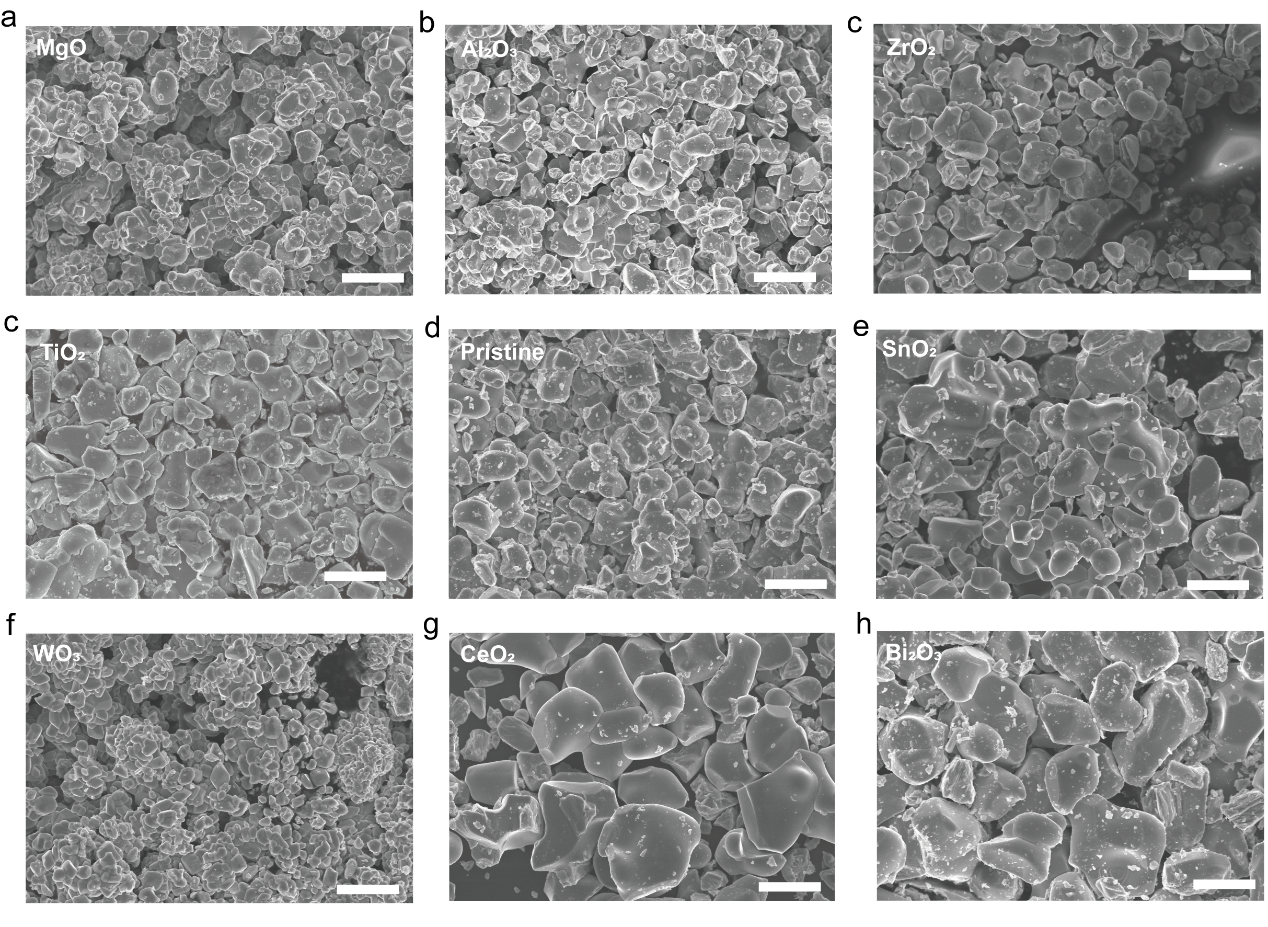


**Figure S2.** Influence of different sintering aids on grain growth**. a-h,** The morphology of SCNR cathodes calcined with different sintering aids. Al_2_O_3_ and MgO that are easily incorporated into the crystal lattice have little effect on the growth of particles. WO_3_ has a significant inhibitory effect on the growth of particles. TiO_2_, ZrO_2_, SnO_2_, CeO_2_ and Bi_2_O_3_ can promote the growth of particles, especially CeO_2_ and Bi_2_O_3_. The atomic ratio of all sintering aids is less than 1%. Scale bars: 10 μm.


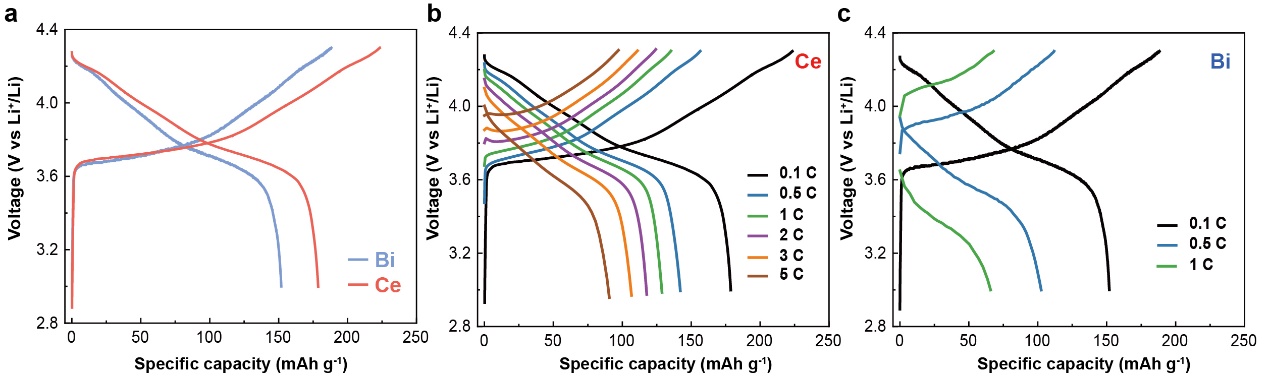


**Figure S3.** Electrochemical performance comparison of samples calcined with sintering aids CeO_2_ or Bi_2_O_3_. **a,** The initial charge-discharge curves. The charge-discharge curves at different current densities for sintering aids **b,** CeO_2_ and **c,** Bi_2_O_3_.


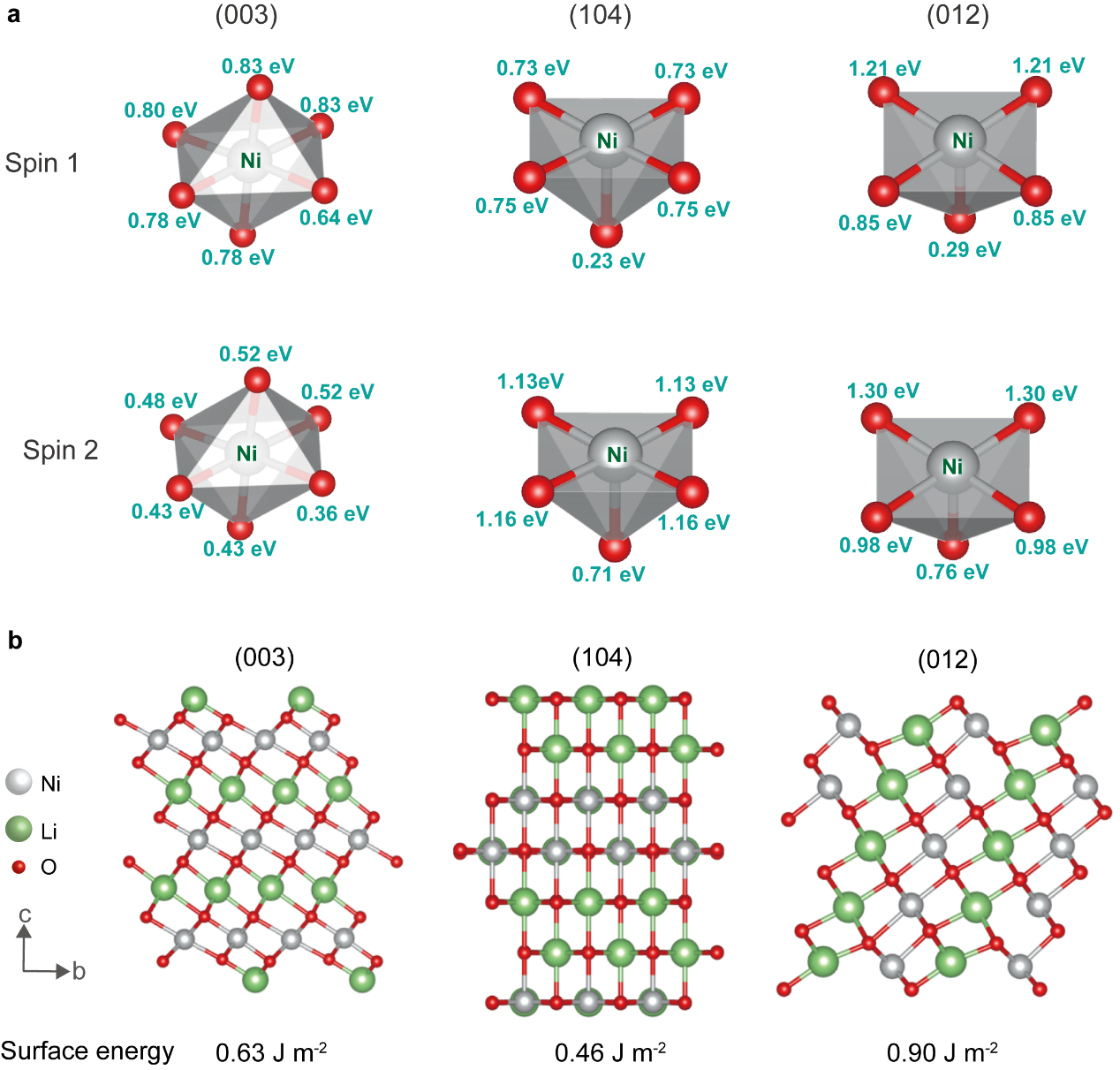


**Figure S4.** First Principles Calculations. a, The bond strength between surface transition metal Ni and oxygen atoms, obtained by crystal orbital Hamilton population (COHP) analysis. **b,** The surface energy of typical exposed facets in Ni-rich cathode materials (003), (104) and (012).


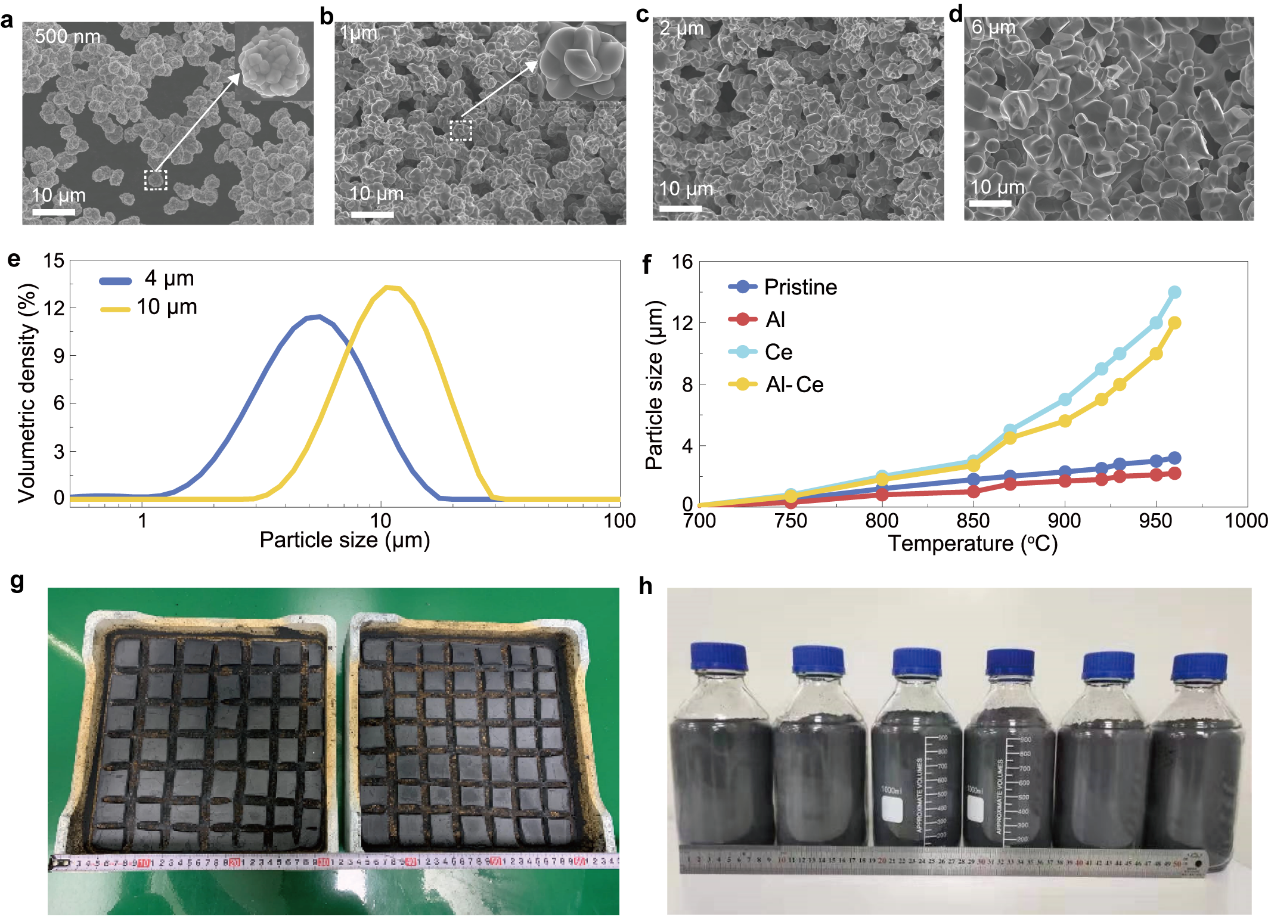


**Figure S5.** Morphology and structure of SCNR cathodes. Morphology of SCNR cathodes with different particle sizes of **a,** 500 nm, **b,** 1 μm, **c,** 2 μm and **d,** 6 μm. **e,** Size distribution of SCNR cathodes. D50 represents the medium value of the particle size distribution. **f,** The plot of the particle size of SCNR cathodes synthesized at different temperatures and with different sintering aids. **g,h,** Photos of SCNR cathodes with a particle size of 10 μm prepared in Kg level.


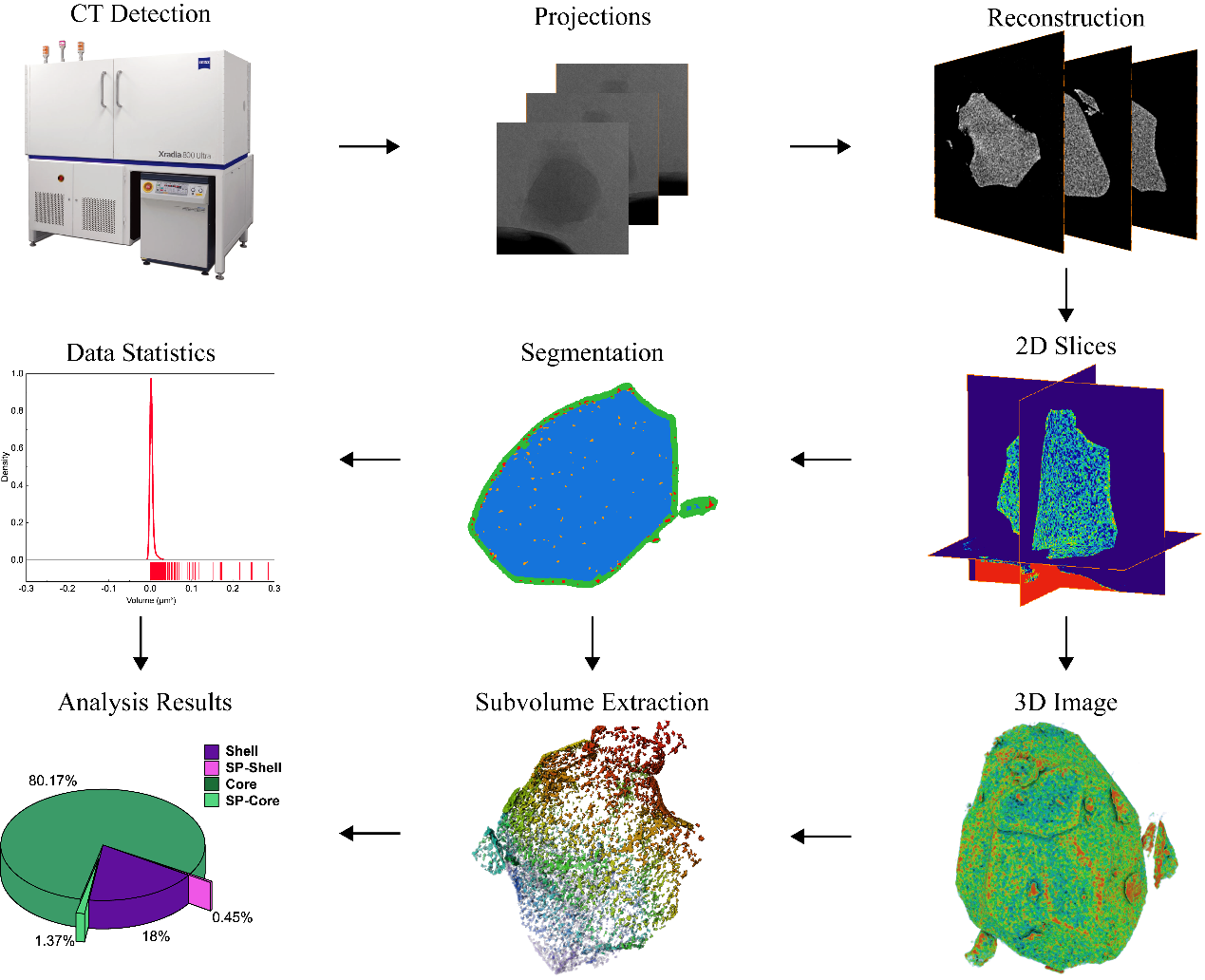


**Figure S6.** The Nano-CT data collection and analysis process. First the CT detection produces a series of two-dimensional projection images, which are used to reconstruct the three-dimensional tomography. Then two-dimensional CT images of different homographs can be obtained. The image segmentation is performed to obtain the desired area ROI, the two-dimensional contrast image and the three-dimensional image of the sample. The threshold segmentation method can acquire different features in the ROI. The post-data statistical analysis of the processed image can be carried out to obtain a quantitative result.


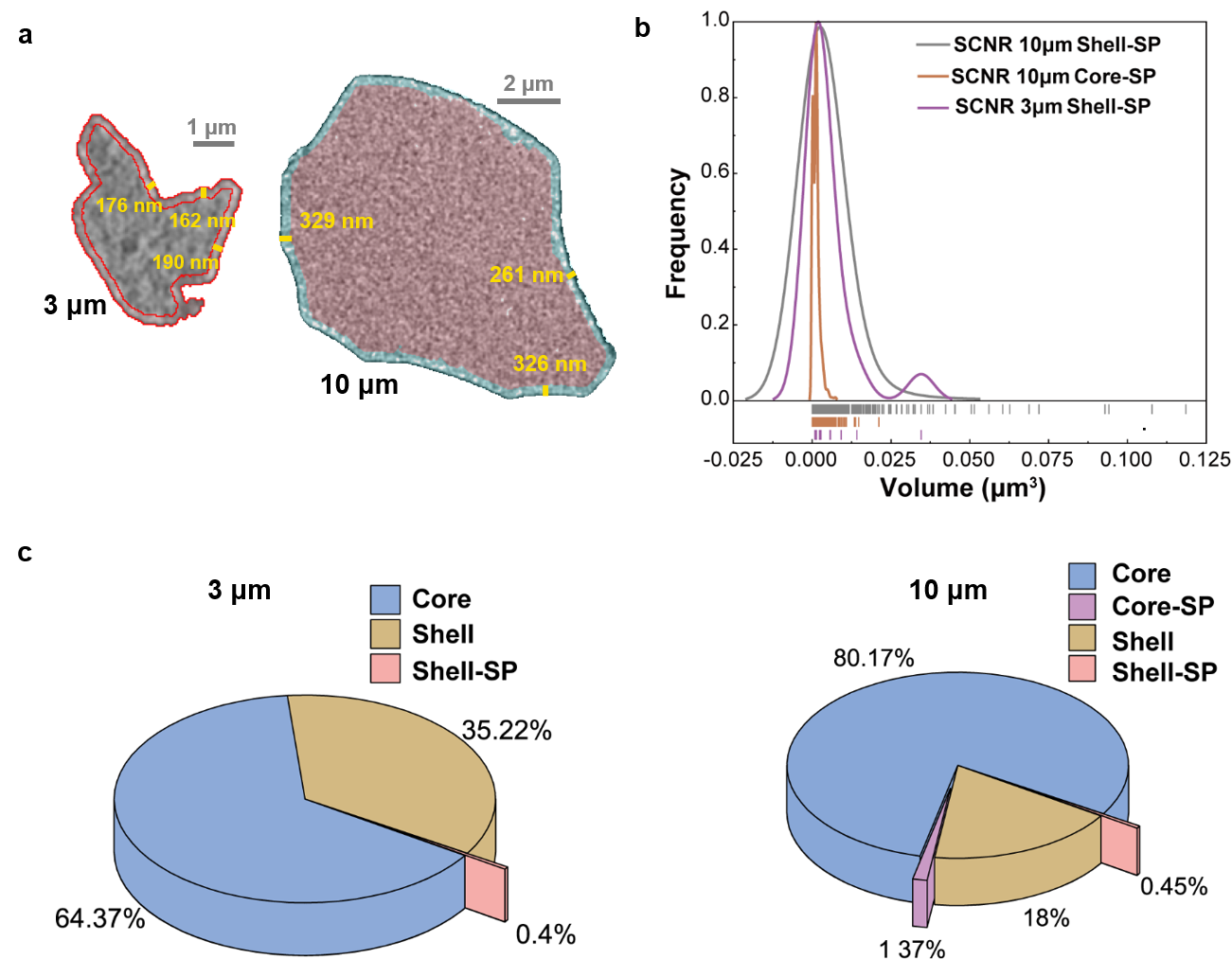


**Figure S7.** The spatial distribution of the trace sintering aids Ce**. a,** 2D slices of the manually divided shell structure of SCNR particles (3 and 10 μm) probed by Nano-CT. **b,** Distribution of the volume of the segregation phases in shell and core regions of SCNR particles. The segregation phase in 10 μm particle shell has a larger average volume of 2.05×10^-3^ μm^3^ and a wider volume distribution than those of 3 μm particles, which is 1.68×10^-3^ μm^3^, demonstrating its densely covered morphology. **c,** Volume ratios of different phases in shell and core regions. The volume ratios of the surface segregation phase in the shell are about 0.4% and 0.45% for 3 μm and 10 μm particles respectively.


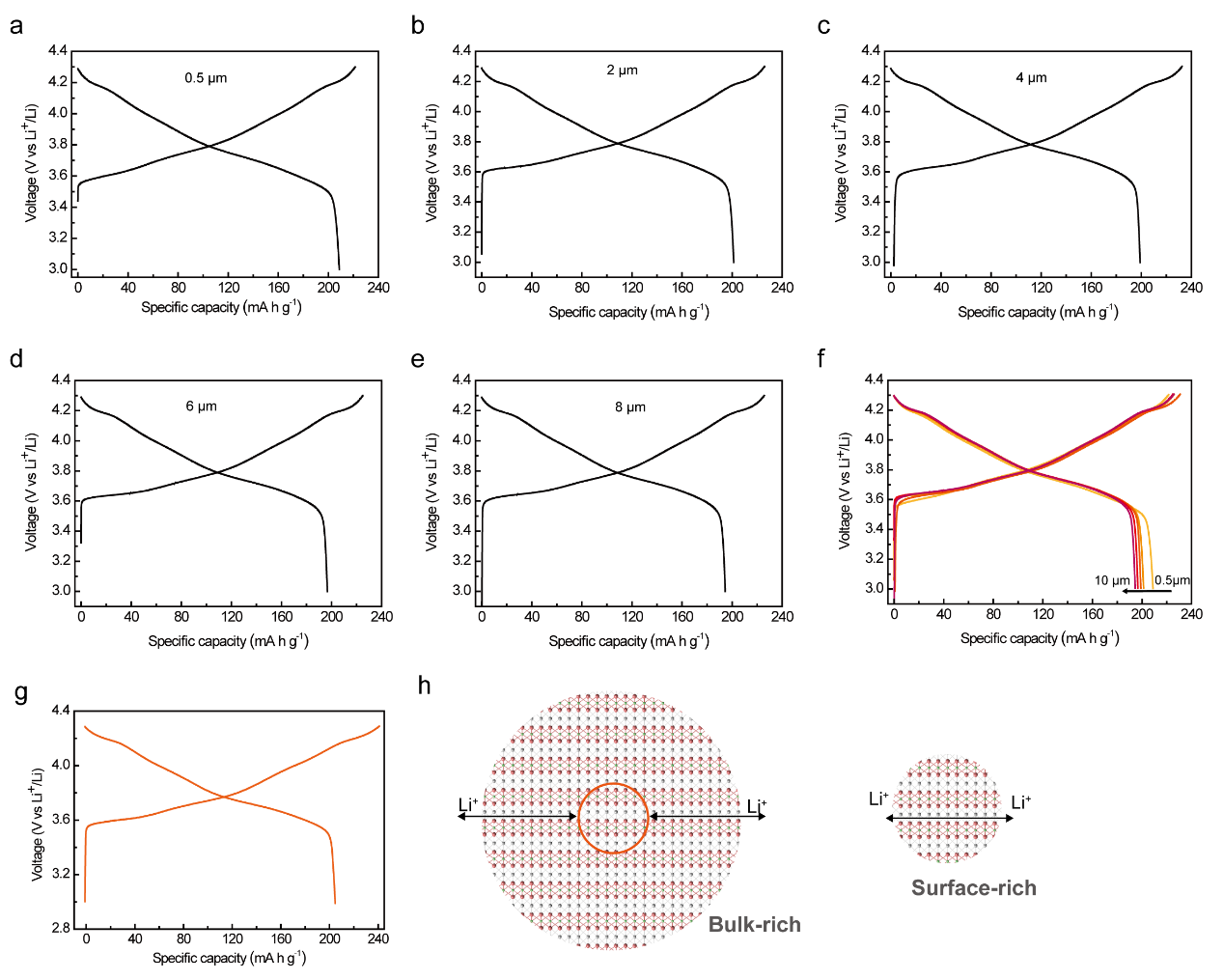


**Figure S8.** Electrochemical performances of SCNR cathodes. **a-e,** Charge-discharge curves of the first cycle of SCNR cathode materials with different particle sizes. **f,** Comparison graph of charge-discharge curves of the first cycle of SCNR cathode materials with different particle sizes. **g,** Charge-discharge curves of the first cycle of SCNR cathode material with particle size 10 μm. The voltage range is 3.0-4.3 V and current C-rate used is 0.01C. **h,** Schematic diagram of the difference of SCNR cathode materials with different particle sizes. Large crystal materials are bulk-rich and small crystal materials are surface-rich. The larger the particle size is, the longer migration path of lithium ions is, which resulting in a slower kinetics.^1-3^


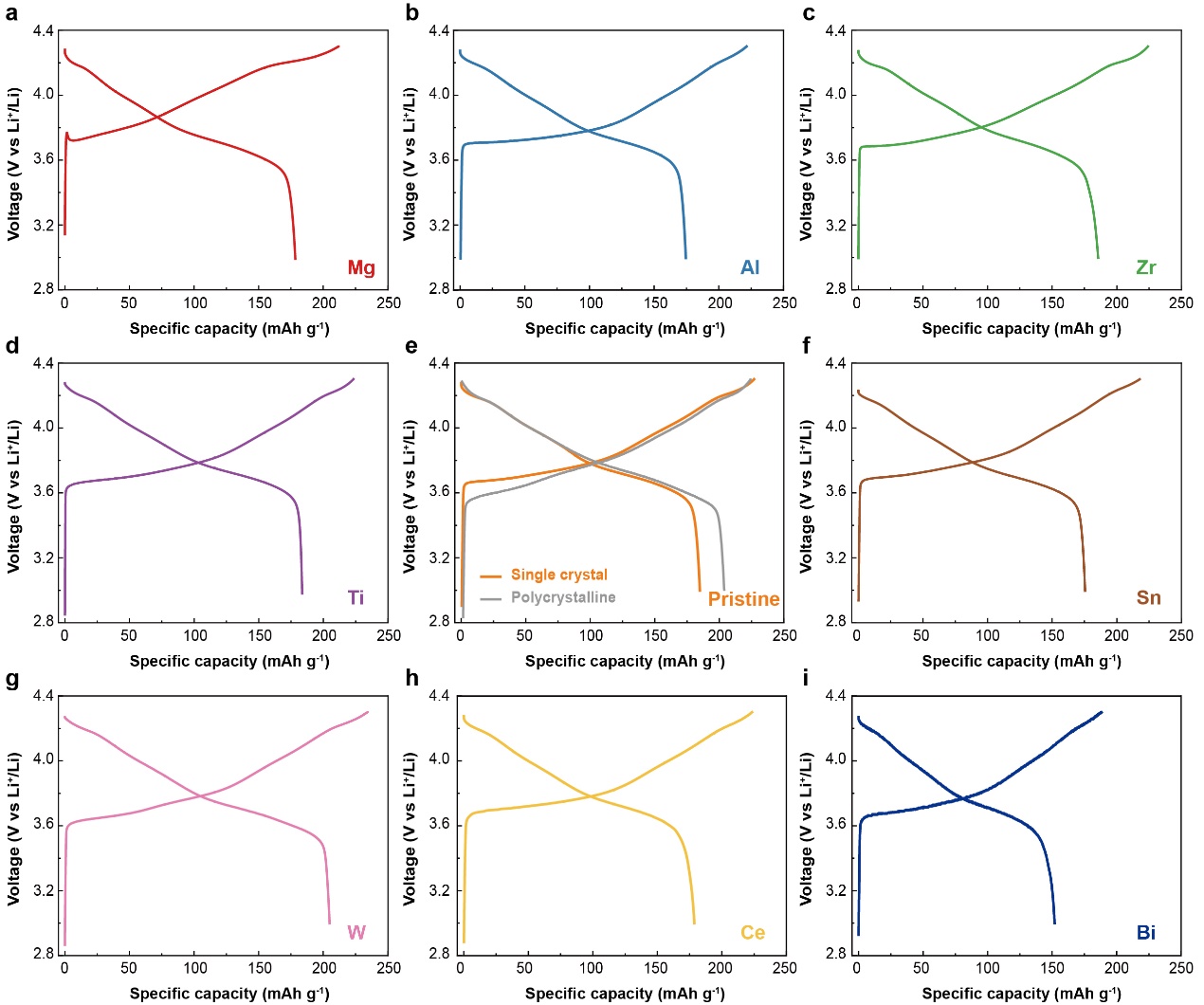


**Figure S9.** The comparison of the electrochemical performance for the cathodes synthesized with different sintering aids. The first charge-discharge curves of the single crystals with sintering aids elements of **a,** Mg, **b,** Al, **c,** Zr, **d,** Ti, **f,** Sn, **g,** W, **h,** Ce, **i,** Bi, and **e,** pristine single-crystal and polycrystalline cathodes.


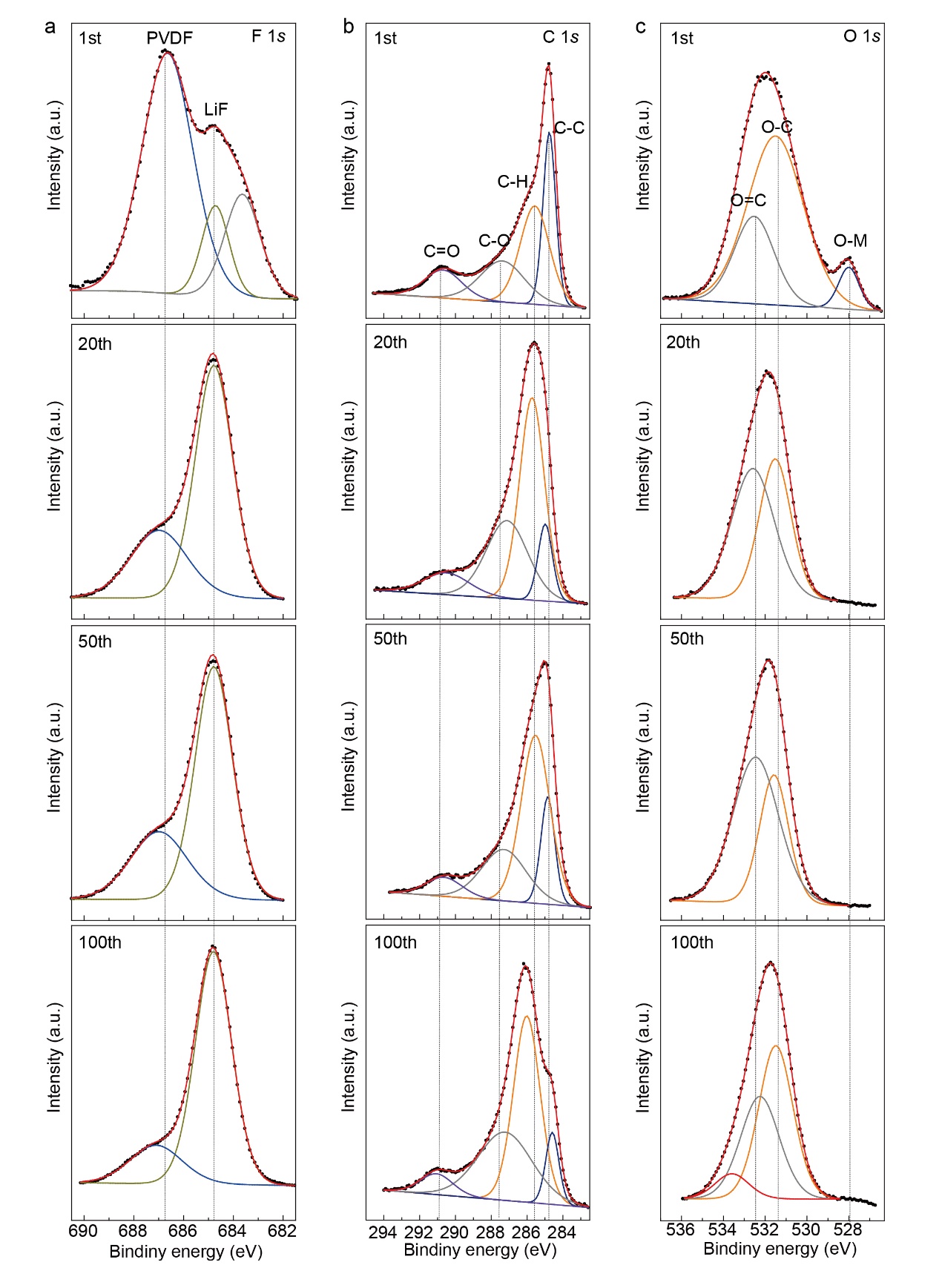


**Figure S10.** XPS analysis of the SCNR electrodes with different charge-discharge cycles. XPS spectra of **a,** F 1*s*, **b,** C 1*s* and **c,** O 1*s*.

**
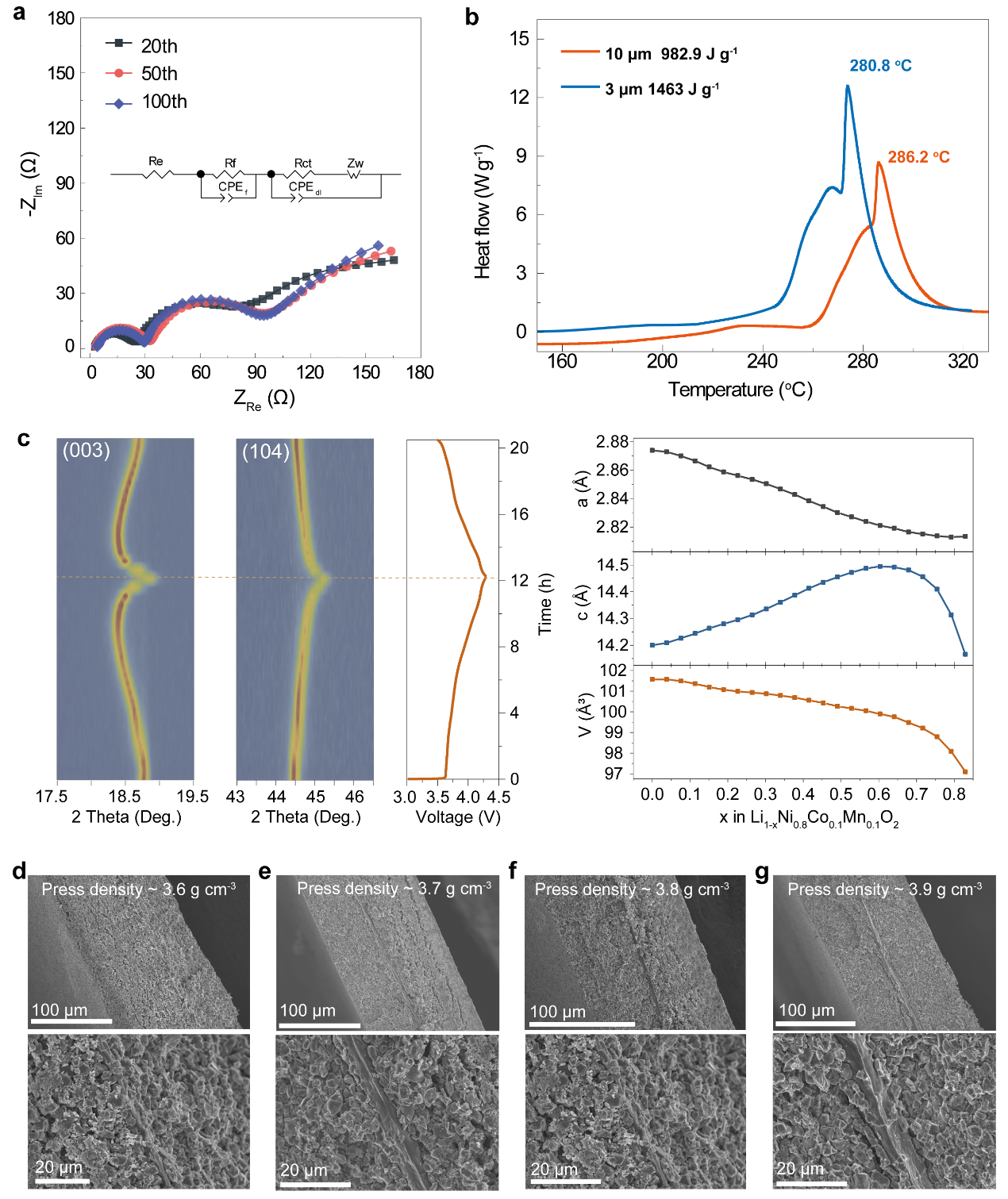
**

**Figure S11.** Interface and structural stability of SCNR cathodes and SEM images of SCNR electrodes. **a,** Impedance spectrum evolution of the 10 μm SCNR cathode. **b,** DSC curves of delithiated SCNR cathodes with the presence of an electrolyte. **c,** *In-situ* XRD patterns of the 10 μm SCNR cathode during the first charge-discharge process. SEM images of the electrodes with press densities of **d,** 3.6 g cm^-3^, **e,** 3.7 g cm^-3^, **f,** 3.8 g cm^-3^ and **g,** 3.9 g cm^-3^. All tests are based on the electrodes after 48 hours of storage.

**Table S1** Summary of single-crystalline Ni-rich NCM cathodes reported in literature.

| **Composition** | **Capacity (mAh g^-1^)** | **Rate** | **Voltage (V)** | **Particle size (µm)** | **Ref** |
| --- | --- | --- | --- | --- | --- |
| LiNi_0.6_Mn_0.2_Co_0.2_O_2_ | 184.2 | 0.1C | 4.5 | 2.5 | 4 |
| LiNi_0.8_Co_0.1_Mn_0.1_O_2_ | 203.9 | 0.1C | 4.3 | 3-6 | 5 |
| LiNi_0.6_Co_0.1_Mn_0.3_O_2_ | 151 | 1C | 4.3 | 2-5 | 6 |
| LiNi_0.7_Co_0.1_Mn_0.2_O_2_ | 195 | 0.1C | 4.4 | 2 | 7 |
| LiNi_0.83_Co_0.11_Mn_0.06_O_2_ | 192.4 | 1C | 4.4 | 4 | 8 |
| LiNi_0.9_Co_0.05_Mn_0.05_O_2_ | 218 | 0.1C | 4.3 | 1 | 9 |
| LiNi_0.76_Mn_0.14_Co_0.1_O_2_ | 196.8 | 0.1C | 4.4 | 3 | 10 |
| LiNi_0.6_Mn_0.2_Co_0.2_O_2_ | 183 | 0.1C | 4.3 | 2 | 11 |
| LiNi_0.8_Co_0.15_Al_0.05_O_2_ | 174 | 0.1C | 4.3 | 2 | 12 |
| LiNi_0.88_Co_0.09_Al_0.03_O_2_ | 192 | 0.2C | 4.3 | 2-5 | 13 |
| LiNi_0.5_Mn_0.3_Co_0.2_O_2_ | 177.5 | 1C | 4.4 | 3-6 | 14 |
| LiNi_0.5_Co_0.2_Mn_0.3_O_2_ | 208 | 0.1C | 4.5 | ~2 | 15 |
| LiNi_0.6_Mn_0.2_Co_0.2_O_2_ | 183 | 0.1C | 4.3 | 4 | 16 |
| LiNi_0.6_Mn_0.2_Co_0.2_O_2_ | 190.0 | 0.1C | 4.4 | 6 | 17 |
| LiNiO_2_ | 196 | 0.1C | 4.3 | 3 | 18 |
| LiNi_0.5_Mn_0.3_Co_0.2_O_2_ | 159.8 | 0.1C | 4.3 | 5 | 19 |
| **SCNR (This work )** | **205** | **0.01C** | **4.3** | **10** | **/** |

**Table S2** The parameters of 4 Ah pouch cells with SiO_x_&graphite as the anode and SCNR as the cathode materials.

| **Cell number** | **Thick**  **(mm)** | **Width***  **Length**  **(mm)** | **Weight**  **(g)** | **Capacity**  **(mAh)** | **Energy**  **(mWh)** | **Volumetric energy**  **(Wh L^-1^)** | **Gravimetric energy**  **(Wh Kg^-1^)** |
| --- | --- | --- | --- | --- | --- | --- | --- |
| 1 | 4.076 | 62*82 | 50.31 | 4232 | 15219 | 734 | 303 |
| 2 | 4.112 | 62*82 | 50.54 | 4272 | 15359 | 734 | 304 |
| 3 | 4.14 | 62*82 | 50.72 | 4283 | 15399 | 731 | 304 |
| 4 | 4.104 | 62*82 | 50.59 | 4241 | 15251 | 730 | 301 |
| 5 | 4.146 | 62*82 | 50.78 | 4288 | 15418 | 731 | 303 |
| 6 | 4.12 | 62*82 | 50.62 | 4278 | 15375 | 734 | 303 |
| 7 | 4.112 | 62*82 | 50.44 | 4291 | 15425 | 737 | 305 |
| 8 | 4.095 | 62*82 | 50.33 | 4247 | 15273 | 733 | 303 |
| 9 | 4.131 | 62*82 | 50.72 | 4266 | 15347 | 730 | 302 |
| 10 | 4.098 | 62*82 | 50.49 | 4280 | 15393 | 738 | 304 |
| 11 | 4.129 | 62*82 | 50.68 | 4278 | 15386 | 732 | 303 |
| 12 | 4.123 | 62*82 | 50.76 | 4293 | 15445 | 737 | 304 |
| 13 | 4.127 | 62*82 | 50.70 | 4274 | 15368 | 732 | 303 |
| 14 | 4.114 | 62*82 | 50.55 | 4291 | 15422 | 737 | 305 |
| 15 | 4.115 | 62*82 | 50.53 | 4262 | 15321 | 732 | 303 |
| 16 | 4.099 | 62*82 | 50.22 | 4237 | 15239 | 731 | 303 |
| 17 | 4.079 | 62*82 | 50.00 | 4220 | 15177 | 731 | 303 |
| 18 | 4.121 | 62*82 | 50.29 | 4242 | 15250 | 727 | 303 |
| 19 | 4.18 | 62*82 | 50.83 | 4295 | 15434 | 726 | 303 |
| 20 | 4.102 | 62*82 | 50.65 | 4280 | 15388 | 737 | 303 |

**Table S3** Key parameters of ARC test of pouch cells under the same volumetric energy density.

| **Material** | **T_1_ (℃)** | **T_2_ (℃)** | **t_1_ (min)** | **t_2_ (min)** | **Δ_t_ (min)** |
| --- | --- | --- | --- | --- | --- |
| LCoO_2_ | 72 | 146 | 342 | 1196 | 854 |
| SCNR | 92 | 157 | 494 | 829 | 335 |
| NCM811 | 83 | 136 | 415 | 711 | 296 |

**Table S4** Samsung SDI’s cell parameters for mobile phone. The data come from the company's official website.

| **Cell Model** | **Thick**  **(mm)** | **Width**  **(mm)** | **Height**  **(mm)** | **Capacity**  **(mA h)** | **Voltage**  **(V)** | **Energy**  **(Wh)** | **Energy density**  **(Wh L^-1^)** | **Ref.** |
| --- | --- | --- | --- | --- | --- | --- | --- | --- |
| PGF3457A4 | 3.4 | 57 | 104 | 2,880 | 3.8 | 10.94 | 543 | 20 |
| PGF364981 | 3.6 | 46 | 81 | 1,950 | 3.7 | 7.22 | 538 | 20 |
| PGF435573 | 4.3 | 55 | 73 | 2,520 | 3.8 | 9.58 | 555 | 20 |
| PGF444872 | 4.4 | 48 | 72 | 2,170 | 3.8 | 8.25 | 543 | 20 |
| PGF445560 | 4.4 | 55 | 60 | 2,050 | 3.8 | 7.79 | 537 | 20 |
| PGF455369 | 4.5 | 53 | 69 | 2,320 | 3.8 | 8.82 | 536 | 20 |
| PGF505858 | 5 | 58 | 58 | 2,370 | 3.8 | 9.01 | 536 | 20 |

**Table S5** A detailed comparison of the electrochemical performance, price and other key parameters of SCNR cathode and commercialized secondary particle NCM811 and LiCoO_2_. The data are provided by Beijing IA metal New Energy Technology Co., LTD. and Dongguan TAFEL New Energy Technology Co., LTD.

| **Material** | **LiCoO_2_** | **NCM811** | **SCNR (This work)** |
| --- | --- | --- | --- |
| **Volumetric energy**  **(Wh L^-1^)** | 2800 | 2500 | 3000 |
| **Gravimetric energy**  **(Wh Kg^-1^)** | 700 | 780 | 760 |
| **Rate capacity**  **(80%)** | 3C | 3C | 3C |
| **Cycle Life**  **(80%)** | 800 | 1200 | 1000 |
| **Safety**  **(DSC-test, ℃)** | 280 | 220 | 280 |
| **Price**  **(US$ per tonne)** | 60000 | 45000 | 45000 |
| **Tap density**  **(g cm^-3^)** | 3.3 | 2.2 | 3.2 |
| **Electrode Press density**  **(g cm^-3^)** | 4.1 | 3.4 | 3.9 |

**Table S6** The main raw material cost of commercialized NCM811 and LiCoO_2_ cathode materials (According to the average market price in August, 2022).

| **Material** | **US$ per tonne** | **LiCoO_2_ (US$ per tonne )** | **NCM811(US$ per tonne )** |
| --- | --- | --- | --- |
| Co_3_O_4_ | 34500 | 28290 (0.82 tonne) | / |
| Li_2_CO_3_ | 72400 | 28960 (0.4 tonne) | / |
| NiSO_4_·6H_2_O | 5500 | / | 11880 (2.16 tonne) |
| MnSO_4_·H_2_O | 910 | / | 157 (0.173 tonne) |
| CoSO_4_·7H_2_O | 7900 | / | 2283 (0.289 tonne) |
| LiOH·H_2_O | 69600 | / | 31320 (0.45 tonne) |
| LiCoO_2_ | / | 57250 | / |
| NCM811 | / | / | 45640 |

**References**

1 Li, W., Asl, H. Y., Xie, Q. & Manthiram, A. Collapse of LiNi_1–x–y_Co­Mn_y_O_2_ Lattice at Deep Charge Irrespective of Nickel Content in Lithium-Ion Batteries. *J. Am. Chem. Soc.* 2019; **141**: 5097-5101.

2 Singer, A. et al. Nucleation of dislocations and their dynamics in layered oxide cathode materials during battery charging. *Nat. Energy* 2018; 3: 641-647.

3 Wang, X., Ding, Y. L., Deng, Y. P. & Chen, Z. Ni‐Rich/Co‐Poor Layered Cathode for Automotive Li‐Ion Batteries: Promises and Challenges. *Adv. Energy Mater.* 2020; **10**: 1903864.

4 Huang, B. *et al.* The effects of reheating process on the electrochemical properties of single crystal LiNi_0.6_Mn_0.2_Co_0.2_O_2_. *Solid State Ionics* 2020; **345**: 115200.

5 Chen, X., Tang, Y., Fan, C. L. & Han, S. C. A highly stabilized single crystalline nickel-rich LiNi_0.8_Co_0.1_Mn_0.1_O_2_ cathode through a novel surface spinel-phase modification. *Electrochim. Acta* 2020; **341**: 136075.

6 Zhang, Z. *et al.* A low cost single-crystalline LiNi_0.60_Co_0.10_Mn_0.30_O_2_ layered cathode enables remarkable cycling performance of lithium-ion batteries at elevated temperature. *J. Power Sources* 2021; **503**: 230028.

7 Cheng, L. *et al.* Al-doping enables high stability of single-crystalline LiNi_0.7_Co_0.1_Mn_0.2_O_2_ lithium-ion cathodes at high voltage. *Rsc Adv.* 2021; **11**: 124-128.

8 Fan, X. M. *et al.* Crack-free single-crystalline Ni-rich layered NCM cathode enable superior cycling performance of lithium-ion batteries. *Nano Energy* 2020; **70**: 104450.

9 Zou, Y. *et al.* Enhanced Cycle Life and Rate Capability of Single-Crystal, Ni-Rich LiNi_0.9_Co_0.05_Mn_0.05_O_2_ Enabled by 1,2,4-1H-Triazole Additive. *ACS Appl. Mater. Interfaces* 2021; **13**: 16427-16436.

10 Bi, Y. *et al.* Reversible planar gliding and microcracking in a single-crystalline Ni-rich cathode. *Science* 2020; **370**: 1313-1317.

11 Qian, G. N. *et al.* Single-crystal nickel-rich layered-oxide battery cathode materials: synthesis, electrochemistry, and intra-granular fracture. *Energy Storage Mater* 2020; **27**: 140-149.

12 Fantin, R. *et al.* Synthesis and Postprocessing of Single-Crystalline LiNi_0.8_Co_0.15_Al_0.05_O_2_ for Solid-State Lithium-Ion Batteries with High Capacity and Long Cycling Stability. *Chem. Mater.* 2021; **33**: 2624-2634.

13 Li, H. *et al.* Synthesis of Single Crystal LiNi_0.88_Co_0.09_Al_0.03_O_2_ with a Two-Step Lithiation Method. *J. Electrochem. Soc.* 2016; **166**: A1956-A1963.

14 Fan, X. M. *et al.* Unravelling the influence of quasi single-crystalline architecture on high-voltage and thermal stability of LiNi0.5Co0.2Mn0.3O2 cathode for lithium-ion batteries. *Chem. Eng. J.* 2020; **393**: 124709.

15 Zhang, Q. *et al.* The surface triple-coupling on single crystalline cathode for lithium ion batteries. *Nano Energy* 2021; **86**: 106096.

16 Qian, G. *et al.* Temperature-Swing Synthesis of Large-Size Single-Crystal LiNi_0.6_Mn_0.2_Co_0.2_O_2_ Cathode Materials. *J. Electrochem. Soc.* 2021; **168**: 010534.

17 Li, H., Li, J., Ma, X. & Dahn, J. R. Synthesis of Single Crystal LiNi_0.6_Mn_0.2_Co_0.2_O_2_with Enhanced Electrochemical Performance for Lithium Ion Batteries. *J. Electrochem. Soc.* 2018; **165**: A1038-A1045.

18 Liu, A. *et al.* Synthesis of Co-Free Ni-Rich Single Crystal Positive Electrode Materials for Lithium Ion Batteries: Part I. Two-Step Lithiation Method for Al- or Mg-Doped LiNiO_2_. *J. Electrochem. Soc.* 2021; **168**: 040531.

19 Li, J. *et al.* Synthesis of Single Crystal LiNi_0.5_Mn_0.3_Co_0.2_O_2_for Lithium Ion Batteries. *J. Electrochem. Soc.* 2017; **164**: A3529-A3537.

20 https://www.samsungsdi.com/lithium-ion-battery/it-devices/mobile-phone.html
